# Research into the characteristic molecules significantly affecting liver cancer immunotherapy

**DOI:** 10.3389/fimmu.2023.1029427

**Published:** 2023-02-13

**Authors:** Junhong Chen, Hengwei Jin, Hao Zhou, Xufei Hei, Kai Liu

**Affiliations:** Department of Hepatobiliary and Pancreatic Surgery II, General Surgery Center, The First Hospital of Jilin University, Changchun, China

**Keywords:** liver cancer, immunotherapy, immune checkpoint, M2 macrophages, CDCA7

## Abstract

**Background:**

The past decade has witnessed unprecedented scientific breakthroughs, including immunotherapy, which has great potential in clinical applications for liver cancer.

**Methods:**

Public data were obtained from The Cancer Genome Atlas (TCGA) and International Cancer Genome Consortium (ICGC) databases and analyzed with R software.

**Results:**

The LASSO and SVM-RFE machine learning algorithms identified 16 differentially expressed genes (DEGs) related to immunotherapy, namely, GNG8, MYH1, CHRNA3, DPEP1, PRSS35, CKMT1B, CNKSR1, C14orf180, POU3F1, SAG, POU2AF1, IGFBPL1, CDCA7, ZNF492, ZDHHC22, and SFRP2. Moreover, a logistic model (CombinedScore) was established based on these DEGs, showing an excellent prediction performance for liver cancer immunotherapy. Patients with a low CombinedScore might respond better to immunotherapy. Gene Set Enrichment Analysis showed that many metabolism pathways were activated in patients with a high CombinedScore, including butanoate metabolism, bile acid metabolism, fatty acid metabolism, glycine serine and threonine metabolism, and propanoate metabolism. Our comprehensive analysis showed that the CombinedScore was negatively correlated with the levels of most tumor-infiltrating immune cells and the activities of key steps of cancer immunity cycles. Continually, the CombinedScore was negatively associated with the expression of most immune checkpoints and immunotherapy response-related pathways. Moreover, patients with a high and a low CombinedScore exhibited diverse genomic features. Furthermore, we found that CDCA7 was significantly correlated with patient survival. Further analysis showed that CDCA7 was positively associated with M0 macrophages and negatively associated with M2 macrophages, suggesting that CDCA7 could influence the progression of liver cancer cells by affecting macrophage polarization. Next, single-cell analysis showed that CDCA7 was mainly expressed in prolif T cells. Immunohistochemical results confirmed that the staining intensity of CDCA7 was prominently increased in the nucleus in primary liver cancer tissues compared to adjacent non-tumor tissues.

**Conclusions:**

Our results provide novel insights into the DEGs and factors affecting liver cancer immunotherapy. Meanwhile, CDCA7 was identified as a potential therapeutic target in this patient population.

## Introduction

Liver cancer is a common digestive malignancy, responsible for nearly 8 million new cases annually (1). Many factors have been established to play a role in liver cancer occurrence, such as genomic differences, lifestyle, hepatitis virus, and fat, accounting for the gradual increase in incidence over the years. In some cases, patients with primary liver cancer can benefit from surgery in terms of long-term survival (1). Nevertheless, only 10% of liver cancer patients are indicated for surgical resection (2). For patients with advanced disease, chemotherapy, local ablation, and biological therapies are the main therapeutic modalities (3). Notwithstanding the fact that significant strides have been made in scientific research in recent years, mainstream treatment options exhibit limited efficacy. For instance, long-term use of sorafenib often leads to drug resistance and side effects that limit the drug’s benefits.

It is widely acknowledged that the human immune system normally recognizes and destroys foreign cells, including cancer cells. In the tumor microenvironment (TME), tumor fragment peptides are presented to major histocompatibility complex (MHC) molecules by antigen-presenting cells, thereby starting the killing process (4). However, multidrug resistance to chemotherapy has emerged as a significant challenge. A previous study showed that stem cells could be targeted by immunotherapy to combat resistance to common chemotherapies (5). Furthermore, sorafenib can prevent immunosuppression, increasing the effect of immunotherapy, like PD-1/L1 inhibitor therapy (6). Consequently, the combination of immunotherapy and other common therapies seems promising. Zhao et al. observed a synergistic effect through the combination of immunotherapy and common drugs for liver cancer (7). Moreover, current evidence suggests that PD-1/L1 inhibitors are correlated with a decrease in hepatitis B and C infections (8). Given that virus infection can induce liver cancer recurrence, therapy targeting PD-1/L1 can reduce relapses (8). Nivolumab, a PD-1 inhibitor, has now been approved by the FDA for treating multiple solid cancers, including liver cancer, and has yielded promising results (9). Meanwhile, the combination of ipilimumab (a CTLA-4 inhibitor) and nivolumab has been used in a clinical trial of liver cancer (10). Overall, immunotherapy has great potential for liver cancer treatment. Thus, exploring the biological molecules and mechanisms affecting liver cancer immunotherapy is essential.

Bioinformatics has become a very “hot” cross-disciplinary field for modern researchers, providing the framework for research in drug discovery, assessment, and development (11). Based on open-access data and bioinformatics algorithms, we systematically investigated the underlying molecules affecting liver cancer immunotherapy. Meanwhile, a CombinedScore was established based on the identified molecules, which showed an excellent prediction ability for the patient’s response to immunotherapy. A significant difference in immunotherapy response was observed in patients with high and low CombinedScore, and the underlying biological differences were identified between these two groups.

## Methods

### Data collection

Complete next-generation sequencing information and clinical parameters of 371 patients with HCC were obtained from The Cancer Genome Atlas (TCGA) data portal (https://gdc-portal.nci.nih.gov/). RNA-seq data and clinical information of external validation cohorts (ICGC-FR and-JP) samples were obtained from the ICGC portal (https://dcc.icgc.org/). Patients with complete expression profiles and corresponding clinical information were included in our analysis. The “Sva” package in R software combined data and reduced batch effects. Baseline information on enrolled patients is provided in **Supplementary File 1**.

### Evaluation of the immunotherapy response

Evaluation of liver cancer immunotherapy was performed through the Tumor Immune Dysfunction and Exclusion (TIDE) website (http://tide.dfci.harvard.edu/) (12). Briefly, the normalized gene expression profile was the input file. The “cancer type” parameter was set as “Other”. The “previous immunotherapy” parameter was set as “No”. Based on TIDE analysis, patients were assigned a TIDE score according to gene expression profile, with a score < 0 defined as immunotherapy responders, and patients with a TIDE score > 0 considered as non-responders. In addition, the submap algorithm was applied to evaluate the patient’s response to immunotherapy according to the data set of 47 patients with melanoma (13), performed using the Submap module in GenePattern (https://cloud.genepattern.org/).

### Identification of differentially expressed genes between responders and non-responders

Differentially expressed genes (DEGs) between responders and non-responders were screened by the “limma” R package based on the criteria |log2-fold change (FC)| > 1 and false discovery rate (FDR) < 0.05.

### Optimal variable identification by machine learning algorithms

Two machine learning algorithms, SVM-RFE (Support Vector Machines-Recursive Feature Elimination) and LASSO (Least Absolute Shrinkage and Selection Operator), were utilized to screen the optimal molecule variables between liver cancer immunotherapy responders and non-responders (14). The glm function in R software was utilized for logistic model construction.

### Biological exploration

Gene ontology (GO) and Kyoto Encyclopedia of Genes and Genomes (KEGG) enrichment analyses were conducted using the “clusterProfiler” package and visualized by the “circlize” package (15). Gene Set Enrichment Analysis (GSEA) was utilized to identify biological differences between two specific groups. Chromosomal number variation (CNV) levels were obtained from the TCGA database.

### Estimation of immunological characteristics in the TME

Six methods, namely, TIMER, QUANTISEQ, MCPcounter, EPIC, CIBERSORT, and CIBERSORT-ABS, were applied to assess the levels of tumor-infiltrating immune cells (TIICs). We calculated the activity of seven critical steps of cancer immunity cycles with the ssGSEA method (16). We collected 46 key immune checkpoints (PD-1, PD-L1, CTLA-4, etc.) and evaluated their relationship with the CombinedScore. T-cell inflamed score (TIS) reflecting pre-existing anti-cancer immunity in the TME could predict the response to immune checkpoint blockade (17).

### Single-cell analysis

The evaluation of specific genes at the single-cell level was conducted based on the Tumor Immune Single-cell Hub website (http://tisch.comp-genomics.org/home/) (18).

### Immunohistochemistry staining

Six paired paraffin-embedded primary liver cancer tissues and corresponding adjacent non-tumor tissues were collected at The First Hospital of Jilin University. Immunohistochemistry (IHC) staining was conducted to assess the expression of CDCA7 of the primary liver cancer tissues compared with the corresponding adjacent non-tumor tissues. Briefly, slide-mounted sections were brought to room temperature, dried for 30 min, and then heated at 105°C for 10 min in a citric acid buffer (0.01 M) for antigen retrieval. Hydrogen peroxide (3%) was utilized to inactivate the endogenous enzyme at room temperature for 10 min. Following the blocking step by bovine serum albumin, the primary antibodies against CDCA7 (1:200; Rabbit# abs140624, absin) were applied to incubate the slices overnight at 4°C. After incubation with the secondary antibody at 37°C for 30 min, horseradish peroxidase and diaminobenzidine were used as chromoplast to visualize the immunohistochemical reaction. The slices were re-stained with hematoxylin and sealed with neutral gum. Finally, they were observed with an upright microscope from Leica (Germany) at ×100 and ×400 magnification, respectively.

### Statistical analysis

All statistical analyses were performed in R software. Kaplan–Meier (KM) survival curve was used to compare the prognosis between the two groups. According to the data distribution, Student *t*-tests and Mann–Whitney *U* tests were applied. The receiver operating characteristic (ROC) curve was utilized to assess the prediction performance of identified variables. A *p*-value < 0.05 was statistically significant.

## Results

### DEGs associated with response to immunotherapy

**Figure 1:**
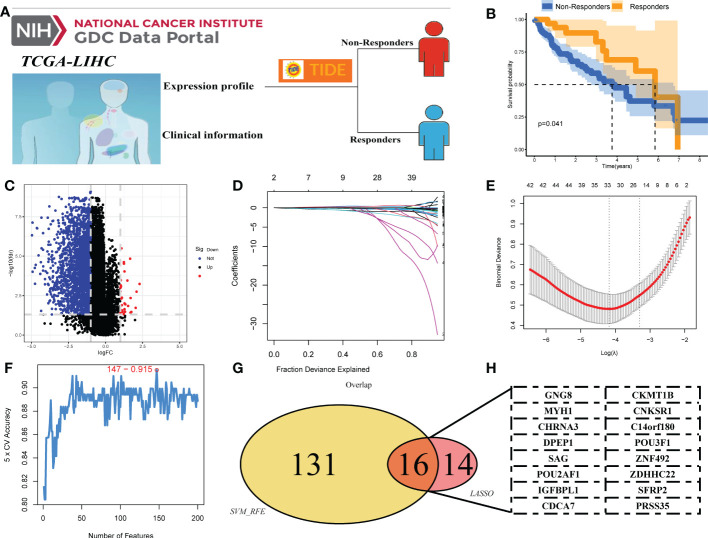
Identification of DEGs of liver cancer immunotherapy. **(A)** TIDE analysis was performed to evaluate the response to immunotherapy in liver cancer patients, with TIDE scores <0 and >0 defining immunotherapy and non-responders, respectively. **(B)** Kaplan–Meier curves between responders and non-responders. **(C)** The volcano plot of DEGs between responders and non-responders. **(D, E)** LASSO algorithm. **(F)** SVM-RFE algorithm. **(G, H)** Sixteen DEGs of immunotherapy were identified, namely, GNG8, MYH1, CHRNA3, DPEP1, PRSS35, CKMT1B, CNKSR1, C14orf180, POU3F1, SAG, POU2AF1, IGFBPL1, CDCA7, ZNF492, ZDHHC22, and SFRP2.

The flowchart of the whole study is shown in **Figure S1**. First, a TIDE analysis was performed, and each patient was assigned a TIDE score, with scores of <0 and >0 defined as immunotherapy responders and non-responders, respectively (**Figure 1A**). KM survival analysis revealed that responders had a superior OS compared to non-responders (log-rank test, *p* = 0.041) (**Figure 1B**). Next, 569 DEGs were identified between responders and non-responders with the following cutoff criteria: |log2FC| > 1 and FDR < 0.05 (**Figure 1C**). We then conducted a functional enrichment analysis to explore the potential biological functions of these DEGs. Significant enrichment in biological processes like passive transmembrane transporter activity and channel activity was found (**Figure S2**). Additionally, KEGG analysis indicated that these DEGs were significantly enriched in cancer-related pathways, including glutamatergic synapse, neuroactive ligand−receptor interaction, and cell adhesion molecules (**Figure S3**). Subsequently, two distinct algorithms, LASSO and SVM-RFE, were utilized to screen the optimal immunotherapy variables. Thirty feature genes were obtained based on the LASSO algorithm to narrow the range of DEGs (**Figures 1D, E**). Meanwhile, we performed the SVM-RFE algorithm on a set of 147 feature genes from the top 200 DEGs based on feature value ranks (**Figure 1F**). After intersecting with genes obtained by the LASSO and SVM-RFE algorithms, 16 candidate genes were identified (GNG8, MYH1, CHRNA3, DPEP1, PRSS35, CKMT1B, CNKSR1, C14orf180, POU3F1, SAG, POU2AF1, IGFBPL1, CDCA7, ZNF492, ZDHHC22, and SFRP2), which were defined as candidate DEGs that determine response to immunotherapy for LIHC (**Figures 1G, H**).

### Performance of differentially expressed genes and logistic model

**Figure 2:**
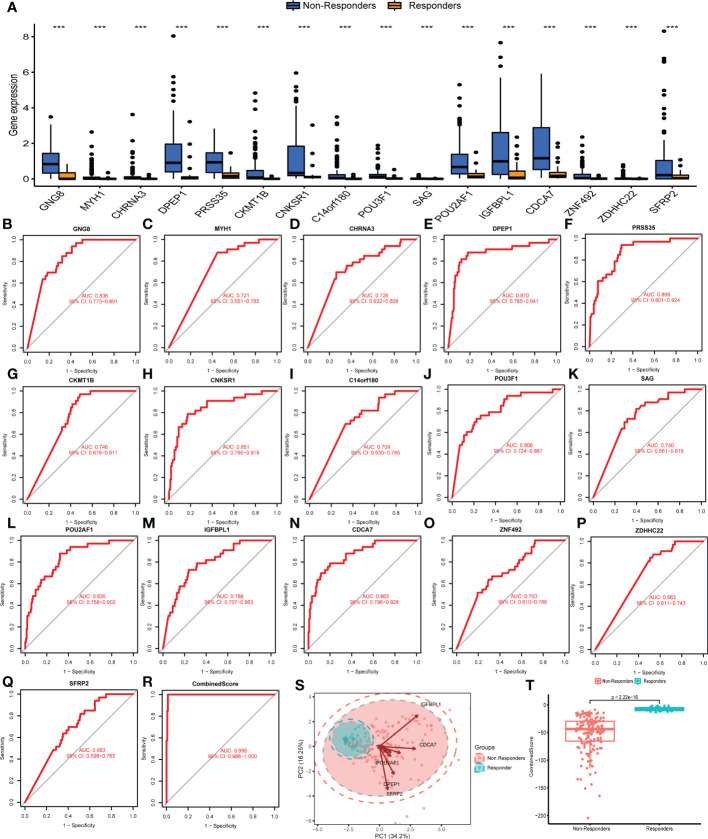
Evaluation of the performance of DEGs. **(A)** Expression level of characteristic genes identified in immunotherapy responders and non-responders. **(B–Q)** ROC curves were utilized to evaluate the prediction ability of characteristic genes in patients’ immunotherapy response. **(R)** The ROC curve of CombinedScore, which was calculated based on the identified characteristic molecules. **(S)** Principal component analysis of the identified genes on patients’ immunotherapy response. **(T)** The CombinedScore difference in immunotherapy responders and non-responders. *** represents *p* < 0.001.

We next compared the difference in these DEGs between immunotherapy responders and non-responders. Results indicated that all DEGs associated with response to immunotherapy, including GNG8, MYH1, CHRNA3, DPEP1, PRSS35, CKMT1B, CNKSR1, C14orf180, POU3F1, SAG, POU2AF1, IGFBPL1, CDCA7, ZNF492, ZDHHC22, and SFRP2, were upregulated in the immunotherapy non-responders (**Figure 2A**). Results of ROC curves showed a good performance for the above DEGs in predicting immunotherapy response (**Figures 2B–Q**; GNG8, AUC value = 0.836; MYH1, AUC value = 0.721; CHRNA3, AUC value = 0.726; DPEP1, AUC value = 0.870; PRSS35, AUC value = 0.868; CKMT1B, AUC value = 0.746; CNKSR1, AUC value = 0.851; C14orf180, AUC value = 0.709; POU3F1, AUC value = 0.808; SAG, AUC value = 0.740; POU2AF1, AUC value = 0.835; IGFBPL1, AUC value = 0.788; CDCA7, AUC value = 0.865; ZNF492, AUC value = 0.703; ZDHHC22, AUC value = 0.683; SFRP2, AUC value =0.683). Moreover, a logistic model was constructed as follows: CombinedScore = GNG8 *−10.78483 + MYH1 *−13.871063 + CHRNA3 *7.145731 + DPEP1 *1.633124 + PRSS35*1.512479 + CKMT1B *−25.128123 + CNKSR1 *−4.046955 + C14orf180 *−11.320222 + POU3F1 *−3.030164 + SAG *−153.590288 + POU2AF1 *2.342797 + IGFBPL1 *−2.818206 + CDCA7 *−1.219284 + ZNF492 *−14.944845 + ZDHHC22 *−98.522042 + SFRP2 *−8.299996. The model yielded an excellent performance in predicting the response to immunotherapy (**Figure 2R**, AUC value = 0.996). Principal component analysis of the 16 DEGs could effectively distinguish between immunotherapy responders and non-responders (**Figure 2S**). Meanwhile, we found that the responders had a higher CombinedScore (**Figure 2T**).

### The calculated CombinedScore is associated with a higher immunotherapy response

**Figure 3:**
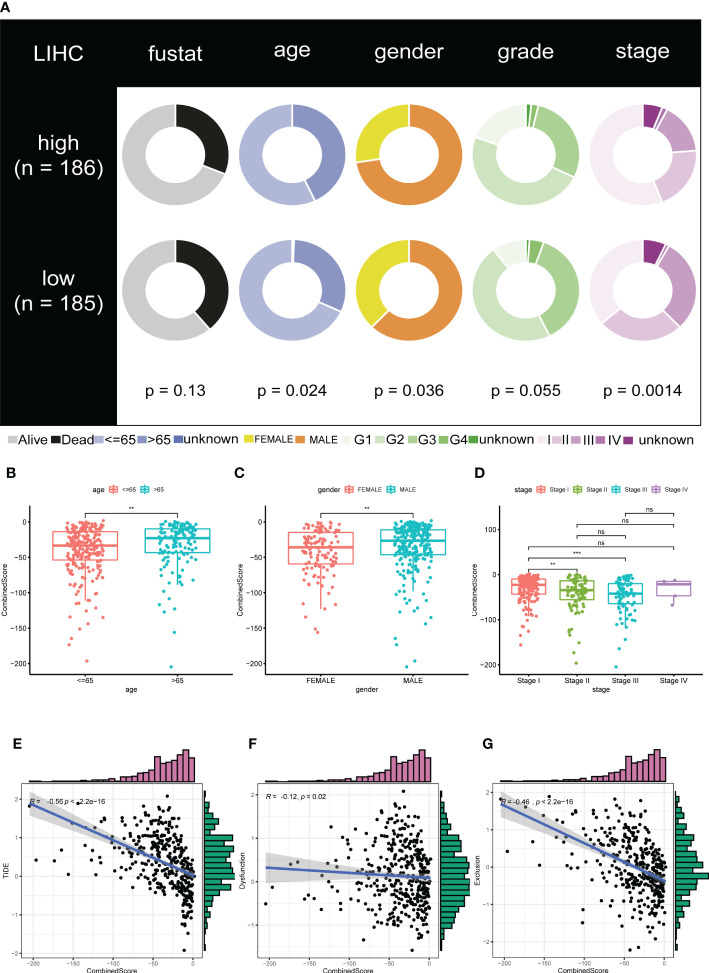
The calculated CombinedScore is associated with clinicopathological features. **(A)** Relationship of the CombinedScore and clinicopathological features in the TCGA-LIHC cohort. **(B–D)** Differences in clinicopathological features, including gender, age, and stage between high- and low-CombinedScore groups. **(E–G)** The CombinedScore had a negative correlation with TIDE score, dysfunction score, and exclusion score. ** represents *p* < 0.01; *** represents *p* < 0.001; NS represents not significant.

**Figure 4:**
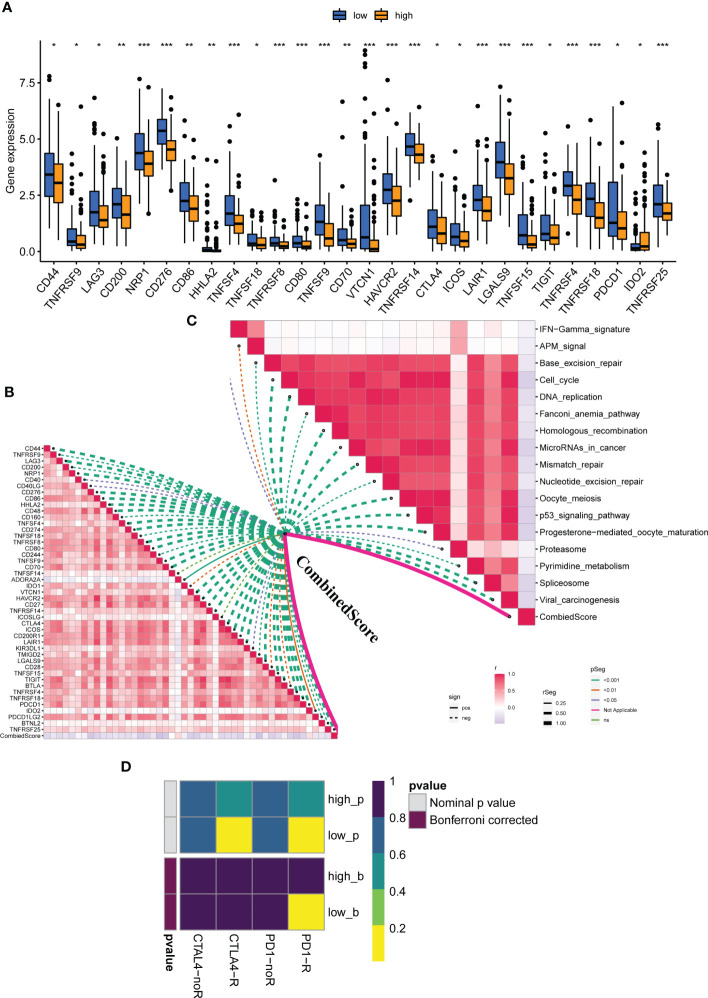
The calculated CombinedScore is associated with patient immunotherapy. **(A)** The key immune checkpoint (PD-1, PD-L1, PD-L2, CTLA-4, etc.) expression in patients with high- and low-CombinedScore in the TCGA-LIHC cohort. **(B)** The correlations between the CombinedScore and the expression of immune checkpoints. **(C)** The correlations between the CombinedScore and scores of immunotherapy-predicted pathways. **(D)** Submap algorithm was conducted to evaluate sensitivity to PD-1 and CTLA-4 blockades in patients with a high- and low-CombinedScore. * represents *p* < 0.05; ** represents *p* < 0.01; *** represents *p* < 0.001.

We examined the relationship between clinical and pathological characteristics and the CombinedScore and found significant differences in clinical parameters, including age, gender, and stage (**Figures 3A–D**). Moreover, we found that the CombinedScore was negatively correlated with the TIDE score, dysfunction score, and exclusion score (**Figures 3E–G**). Considering the difference between patients with a high and low CombinedScore for immunotherapy, we compared the key immune checkpoints among those patients. Interestingly, the results indicated that all immune checkpoint genes (PD-1, PD-L1, CTLA-4, etc.) were significantly upregulated in patients with a low CombinedScore (**Figure 4A**). Furthermore, the CombinedScore was negatively correlated with the expression of all immune checkpoints and the scores of various immunotherapy response-related pathways, including the IFN-γ signature, APM signal, and base excision repair (**Figures 4B, C**). Moreover, the results of the SubMap algorithm revealed that the patients with a low CombinedScore responded better to both PD-1 and CTLA4 blockades (**Figure 4D**).

### Validation of the CombinedScore in the ICGC cohort

**Figure 5:**
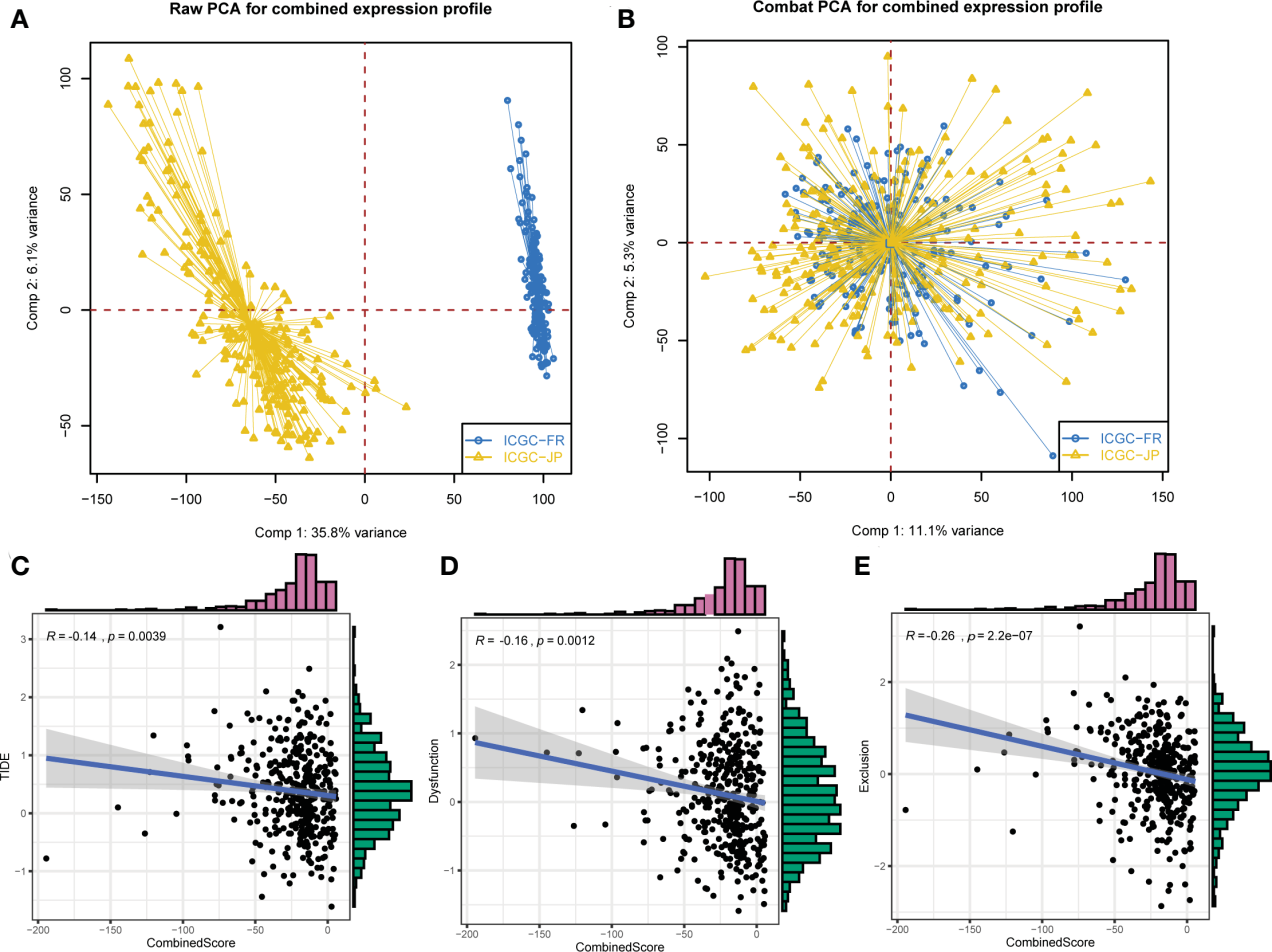
External validation in the ICGC cohort. **(A)** ICGC-FR and ICGC-JP were selected for validation. **(B)** The “Sva” package was used for data combination (ICGC-FR and ICGC-JP). **(C–E)** The CombinedScore had a negative correlation with TIDE score, dysfunction score, and exclusion score in the combined ICGC cohort.

Next, the ICGC cohort was used to validate our results. The ICGC-FR and ICGC-JP were selected, and a significant batch effect was observed. Using the “sva” package in the R software, we performed a combination of data and significantly reduced the batch effect (**Figures 5A, B**). In the combined ICGC cohort, we calculated the CombinedScore for validation. A negative correlation was found between the CombinedScore and the TIDE score, dysfunction score, and exclusion score in the combined ICGC (**Figures 5C–E**). Moreover, all immune checkpoint genes were significantly upregulated in patients with a low CombinedScore (**Figure S4A**). Furthermore, the CombinedScore was negatively related to the expression of key immune checkpoints and the scores of various immunotherapy response-related pathways, like the IFN-γ signature, APM signal, and base excision repair (**Figures S4B, C**). Meanwhile, in the ICGC cohort, we found that the patients with a low CombinedScore were more sensitive to PD-1 and CTLA4 blockades (**Figure S4D**).

### Calculated CombinedScore is associated with liver cancer immune microenvironment

**Figure 6:**
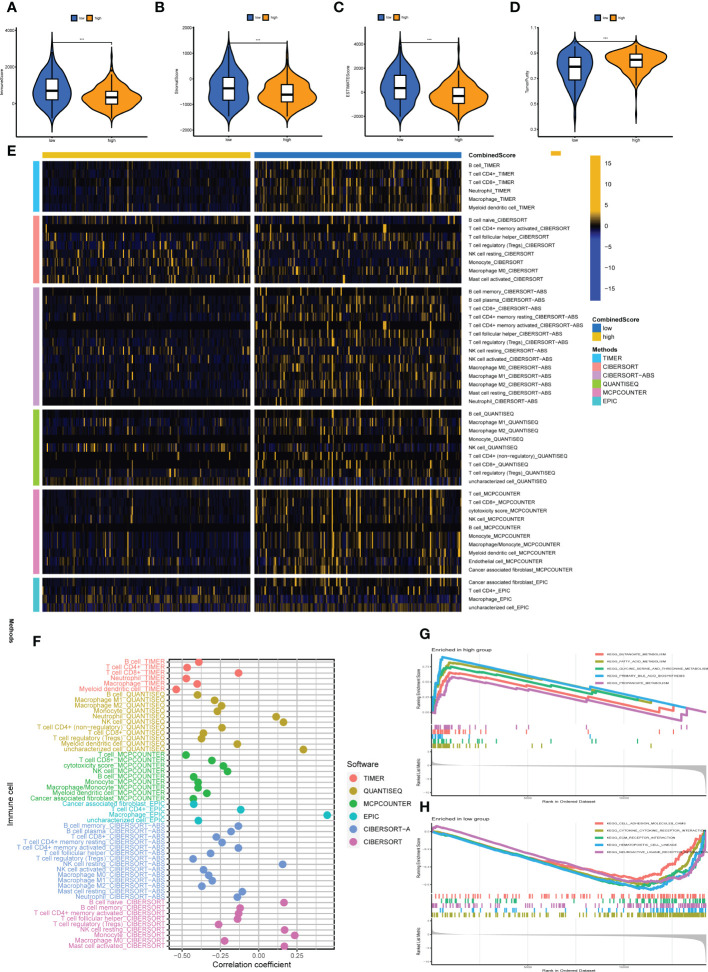
Immune microenvironment analysis. **(A–D)** Comparisons of the stromal score, immune score, ESTIMATE score, and tumor purity in high- and low-CombinedScore groups. **(E)** Heatmap showing immune cell infiltration levels in high- and low-CombinedScore groups by six immune infiltration algorithms (TIMER, CIBERSORT, CIBERSORT-ABS, QUANTISEQ, MCPCOUNTER, and EPIC) was used to quantify the immune microenvironment of liver cancer patients. **(F)** Correlation analysis between the abundance of immune cells infiltrating from six immune infiltration algorithms and the CombinedScore. **(G, H)** Results of the GSEA in high- and low-CombinedScore groups. *** represents *p* < 0.001.

It is well established that a complex immune microenvironment can affect the response to immunotherapy in LIHC patients. We adopted several immune assessment algorithms to investigate the relationship between the CombinedScore and the TME. Interestingly, the ESTIMATE algorithm revealed that the immune score, stromal score, and ESTIMATE score were significantly lower in the high-score group than in the low-score group (**Figures 6A–C**). The high-score group had higher tumor purity (**Figure 6D**). We employed six algorithms (TIMER, CIBERSORT, CIBERSORT-ABS, QUANTISEQ, MCPCOUNTER, and EPIC) to estimate the levels of TIICs. It was found that higher infiltration levels of numerous TIICs (including CD8+ T cells, CD4+ T cells, NK cells, macrophages, and DCs) were concentrated in the low-score group (**Figure 6E**). Spearman correlation analysis further suggested that most recognized TIICs were negatively correlated with the CombinedScore (**Figure 6F**). Furthermore, there were significant negative correlations between the CombinedScore and the activities of the anti-cancer immunity cycles, such as the release of cancer cell antigens, anti-cancer immune priming and activation, and immune cell trafficking (**Figure S5**). Consistently, these findings indicated that anti-cancer activity was higher in patients with lower CombinedScore.

### Biological difference

**Figure 7:**
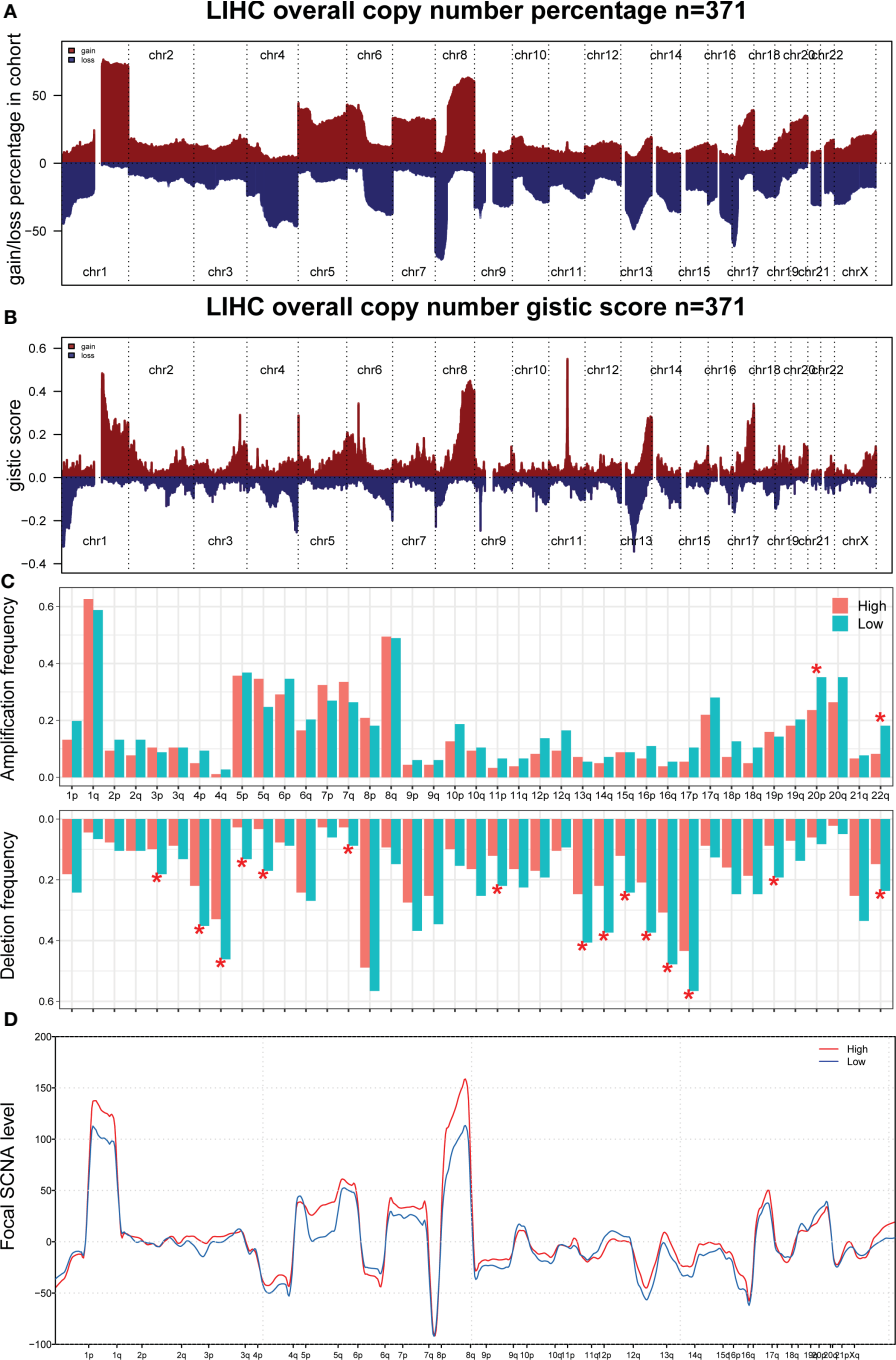
Genomic burden. **(A, B)** The copy number burden of TCGA-LIHC patients in percentage and GISTIC score level. **(C)** The genomic difference between patients with high- and low-CombinedScore in amplification frequency and deletion frequency levels. **(D)** The genomic difference between patients with a high- and low CombinedScore in SCNA level. * represents *p* < 0.05.

Current evidence suggests that biological differences can lead to diverse treatment outcomes. GSEA indicated that in patients with a high CombinedScore, many metabolism pathways were activated, including butanoate metabolism, fatty acid metabolism, glycine serine and threonine metabolism, primary bile acid biosynthesis, and propanoate metabolism (**Figure 6G**). For patients with a low CombinedScore, cell adhesion molecules (cams), cytokine–receptor interaction, ECM–receptor interaction, hematopoietic cell lineage, and neuroactive ligand–receptor interaction were enriched (**Figure 6H**). To further explore genomic differences, we quantified the percentage of copy number and the corresponding GISTIC score of TCGA-LIHC patients (**Figures 7A, B**). Patients with a low CombinedScore had a higher level of frequency of amplification at chromosome 20q and 22q sites and a higher deletion frequency at the 3p, 4p, 4q, 5p, 5q, 7q, 11p, 13q, 14q,15q,16p, 16q, 17p, 19p, and 22q sites (**Figure 7C**). Moreover, there are some genomic differences between patients with a high- and low CombinedScore in SCNA level (**Figure 7D**).

### CDCA7 is associated with patient survival

**Figure 8:**
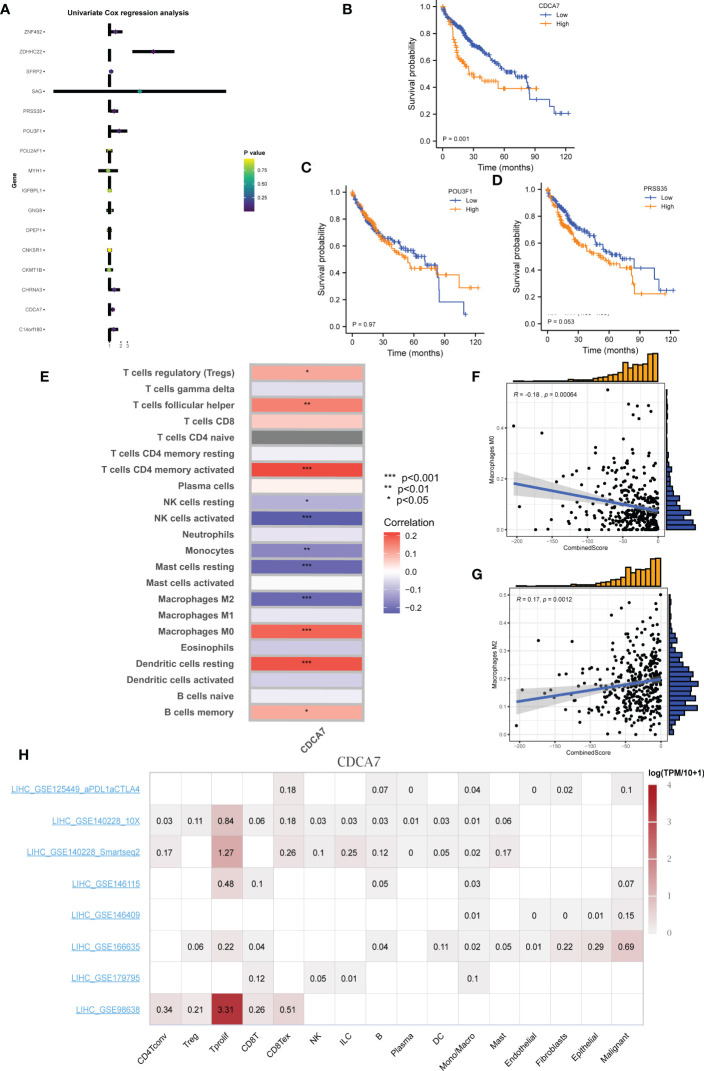
CDCA7 is associated with patients’ survival. **(A)** Univariate Cox analysis of the characteristic molecules. **(B–D)** KM survival curves of CDCA7, POU3F1, and PRSS35. **(E)** Correlation of immune cell infiltration levels with group infiltration levels using the CIBERSORT algorithm. **(F, G)** Correlation of CombinedScore infiltration levels with M0 and M2 macrophage. **(H)** Single-cell analysis of CDCA7 in liver cancer. * represents *p* < 0.05; ** represents *p* < 0.01; *** represents *p* < 0.001.

**Figure 9:**
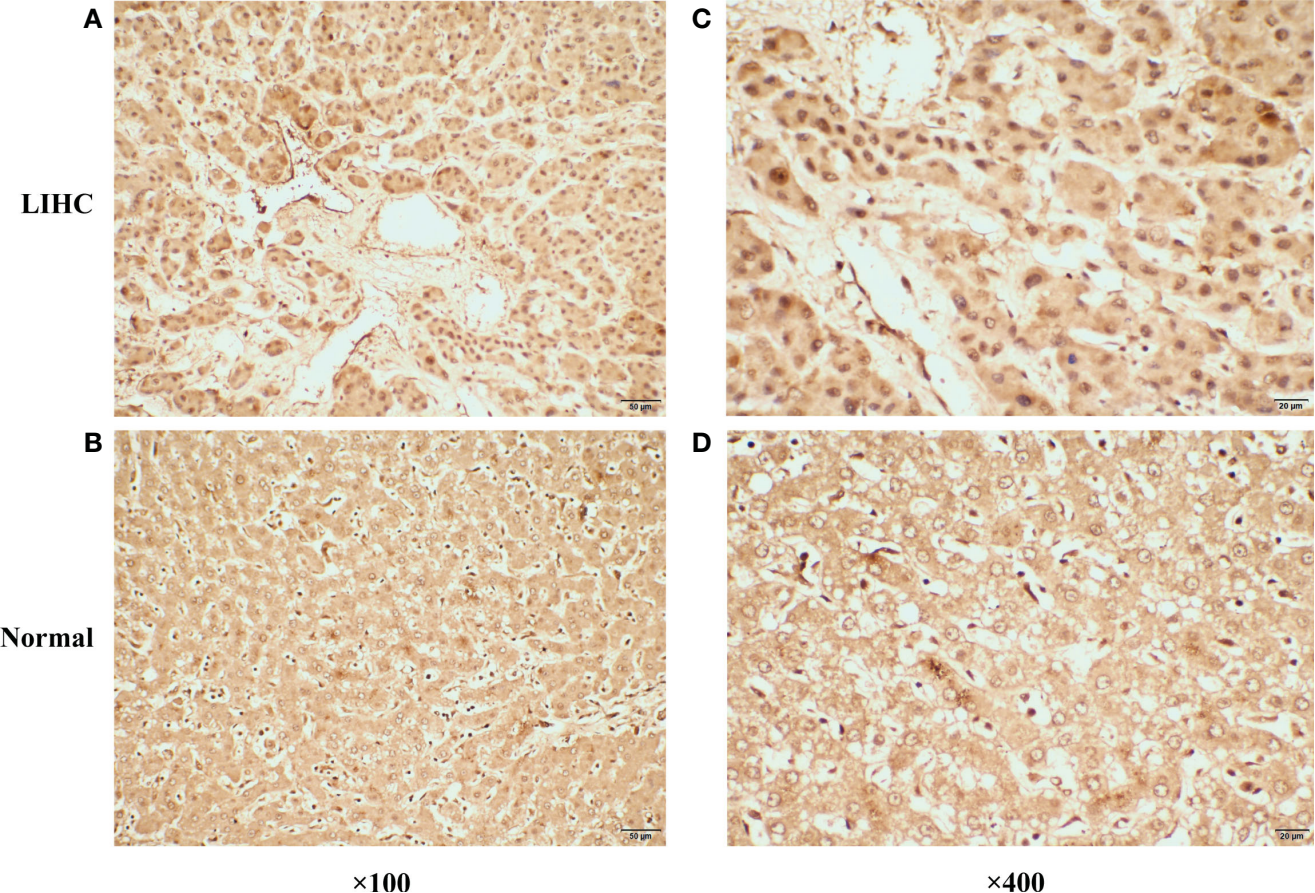
Representative immunohistochemical staining of CDCA7 in primary liver cancer tissues and corresponding adjacent non-tumor tissues. **(A)** The expression of CDCA7 in primary liver cancer tissues (×100). **(B)** The expression of CDCA7 in corresponding adjacent non-tumor tissues (×100). **(C)** The expression of CDCA7 in primary liver cancer tissues (×400). **(D)** The expression of CDCA7 in corresponding adjacent non-tumor tissues (×400).

Univariate Cox and KM survival analyses were conducted based on the identified DEGs, and CDCA7 was significantly correlated with patient survival (**Figures 8A–D**). The correlation analysis indicated that the expression of CDCA7 was positively correlated with Tregs, follicular helper T cells, activated CD4+ memory T cells, resting dendritic cells, and memory B cells, and negatively correlated with resting NK cells, activated NK cells, monocytes, and resting mast cells (**Figure 8E**). We explored the correlation between CDCA7 and macrophages. Moreover, the result of the CIBERSORT algorithm showed that CDCA7 was positively correlated with M0 macrophages but negatively correlated with M2 macrophages (**Figure 8E**). Furthermore, the correlation analysis showed that the CombinedScore was positively correlated with M2 macrophages and negatively associated with M0 macrophages (**Figures 8F, G**). Accordingly, CDCA7 was used for further exploration. Single-cell analysis showed that CDCA7 was expressed mainly in prolif T cells (**Figure 8H**). Furthermore, our immunohistochemical results confirmed that the staining intensity of CDCA7 was prominently increased in the nucleus in primary liver cancer tissues compared to adjacent non-tumor tissues (**Figures 9A–D**).

## Discussion

Liver cancer is the leading cause of cancer-related deaths globally and ranks fifth in the United States (1). The incidence of liver disease in developing countries is higher than in developed countries, making liver cancer one of the deadliest cancers (19). Various dietary exposures, genomic differences, lifestyle, hepatitis virus, and fat are risk factors for liver cancer (20). Current evidence suggests that liver cancer is associated with a poor prognosis, with only 5% to 15% of patients indicated for surgical resection with early-stage disease (21). Consequently, much emphasis has been placed on identifying new treatment options. Interestingly, it is widely believed that combining drugs and altering drug administration/delivery methods can improve malignant tumor outcomes (22). The death of cancer cells is usually mediated by the immune system. MHC molecules of class I and II receive tumor-fragmented peptides during this process (23). Additionally, tumor progression can be prevented by targeting tumor growth biomarkers and connective tissue formation (24). There is no doubt that immunotherapy has great potential for clinical application in liver cancer patients.

Here, we performed a TIDE analysis to evaluate the response rate to immunotherapy in LIHC patients. Based on LASSO logistic regression and SVM-RFE algorithms, we identified GNG8, MYH1, CHRNA3, DPEP1, PRSS35, CKMT1B, CNKSR1, C14orf180, POU3F1, SAG, POU2AF1, IGFBPL1, CDCA7, ZNF492, ZDHHC22, and SFRP2 as DEGs of liver cancer immunotherapy. During clinical practice, detecting the relative expression of these characteristic genes could help clinicians predict the response to liver cancer immunotherapy, guiding individualized therapy. We then constructed a logistic model based on DEGs for immunotherapy, which was also verified in the combined ICGC cohort. Notably, the complex interaction between the TME and tumor cells significantly affects immune escape and immunotherapeutic efficacy (25). Therefore, understanding the TME status and the proportion of immune cell infiltration might contribute to optimizing treatment strategies and assessing tumor prognosis.

We investigated the underlying players of the CombinedScore in terms of TME features. Patients with a lower CombinedScore had higher ESTIMATE, immune, and stromal scores and lower tumor purity. The results of six different immune filtration platforms revealed that patients with a lower CombinedScore had relatively high innate and adaptive TIIC infiltration, while patients with a higher CombinedScore exhibited relatively low TIIC infiltration. As a result, patients with a lower CombinedScore exhibited higher activity of preexisting antitumor immunity in TME.

Interestingly, the results of the TIDE algorithm suggested that the CombinedScore was negatively correlated with the TIDE score and dysfunction score. Indeed, the TIDE score integrated T-cell dysfunction and removal characteristics and simulated tumor-immune escape with different levels of TIICs (26). In many solid tumors, although TIICs significantly infiltrate the tumors, T-cell dysfunction is observed with higher tumor infiltration of macrophages (M2) and the overexpression of many inhibitory immune checkpoints, which could weaken the ability of TIICs to kill tumor cells and promote the growth and progression of tumors, resulting in tumor invasion and metastasis (26, 27). This finding was consistent to a certain extent with our results. We found that the CombinedScore was negatively related to the scores of several immunotherapy-predicted signatures and steps of the cancer-immunity cycle.

Furthermore, our study confirmed that the CombinedScore had a negative relationship with the expression of key immune checkpoints, showing a higher expression in the low CombinedScore group. These results emphasized that although patients with a low CombinedScore had more TIICs in the TME, its immune cell dysfunction and immune escape were also stronger, which could weaken the ability of immune cells to kill tumor cells, consistent with the literature. Due to the higher immunosuppression and lower immunoreactivity in the TME, such patients were often more suitable for immune checkpoint inhibitor therapy (28). We also confirmed that the low CombinedScore group may respond better to immune checkpoint inhibitor treatment using the SubMAP algorithm.

We then performed GSEA to investigate the significant pathways differentially activated between high- and low-CombinedScore groups. GSEA showed that upregulated pathways included butanoate metabolism, fatty acid metabolism, glycine serine and threonine metabolism, primary bile acid biosynthesis, and propanoate metabolism. In most tumors, lipid metabolism is abnormally activated, which allows them to produce, prolong, and desaturate fatty acids to fuel their growth (29). An alteration in cancer cell energy metabolism influences immune responses in the TME, which promote tumor growth (30). It has been shown that PIWIL1 enhances energy production by regulating fatty acid metabolism and inhibits liver cancer progression (31). While elucidating the results of logistic modeling, we also discussed the roles of individual characteristic molecules of immunotherapy, specifically in tumor immunomodulation.

Interestingly, we found that CDCA7 exhibited a negative association with tumor prognosis. A previous study showed that CDCA7 was highly expressed in HCC, which could facilitate cell proliferation and invasion of HCC by recruiting CEBPB to elevate the expression of EZH2 (32). Cai et al. also demonstrated that the downregulation of CDCA7 suppressed EZH2 expression to arrest angiogenesis (33). Moreover, the target gene of Myc CDCA7 is reportedly overexpressed in human cancer and promotes tumor transformation (34, 35). Li et al. indicated that CDCA7 was highly involved in EMT by regulating the expression of Smad4 and Smad7 in the TGF-β signaling pathway (36). Thereafter, we focused on analyzing the specific association between CDCA7 and immunocyte infiltration and found that CDCA7 was negatively correlated with M2 macrophages and positively correlated with M0 macrophages. Our findings suggested that CDCA7 could influence liver cancer cell progression by affecting macrophage polarization, providing a theoretical basis for liver cancer immunotherapy. Future studies are warranted to validate the effects of CDCA7 on tumor biology and macrophage polarization in biological experiments such as cell culture and mouse models.

In summary, based on high-quality data and analysis, our findings provide a foothold for future studies in liver cancer immunotherapy. We developed and validated a well-grounded logistic model according to TIDE results in LIHC, which could effectively predict the immunotherapy response of liver cancer patients. Meanwhile, several limitations and shortcomings were found in this study. First, the findings of our study may be affected to a certain extent by racial bias, given the low proportion of Asians and Africans in the enrolled samples. Furthermore, in some cases, clinical information was incomplete, such as information on M-stage for a large percentage of patients. Finally, it should be borne in mind that the immunotherapy response predicted by TIDE was determined by a bioinformatics algorithm, which exhibits a limited ability to reflect real-world situations. More robust conclusions are expected from studies analyzing the genomic data of LIHC patients treated with immunotherapy in the future.

## Data availability statement

The original contributions presented in the study are included in the article/**Supplementary Material**, further inquiries can be directed to the corresponding author/s.

## Ethics statement

The studies involving human participants were reviewed and approved by the ethics committee of the first hospital of Jilin University. Written informed consent for participation was not required for this study in accordance with the national legislation and the institutional requirements.

## Author contributions

KL and JC contributed to the conception and design of the study. JC and HZ performed data extraction and analysis. JC and HJ wrote the first draft of this manuscript and KL completed the final version of the manuscript. All authors contributed to the article and approved the submitted version.

## Supplementary material

Supplementary Figure 1 The whole flow chart of the study.

Supplementary Figure 2 GO enrichment analyses of DEGs.

Supplementary Figure 3 KEGG enrichment analyses of DEGs.

Supplementary Figure 4
**(A)** Expression of key immune checkpoints (PD-1, PD-L1, PD-L2, CTLA-4, etc.) in patients with a high- and low-CombinedScore in the combined ICGC cohort; **(B)** Correlations between the CombinedScore and expression of immune checkpoints in the combined ICGC cohort; **(C)** Correlations between the CombinedScore and immunotherapy predicted pathways scores in the combined ICGC cohort; **(D)** Submap algorithm was conducted to evaluate the sensitivity to PD-1 and CTLA-4 blockades in patients with a high- and low-CombinedScore in the combined ICGC cohort.

Supplementary Figure 5 Correlation analysis between CombinedScore and the activities of the anticancer immunity cycles.
