# Supplementary figures and images for: Research into the characteristic molecules significantly affecting liver cancer immunotherapy
Source: Front Immunol. 2023 Feb 13;14:1029427. doi: 10.3389/fimmu.2023.1029427 (PMC9968832; doi:10.3389/fimmu.2023.1029427)

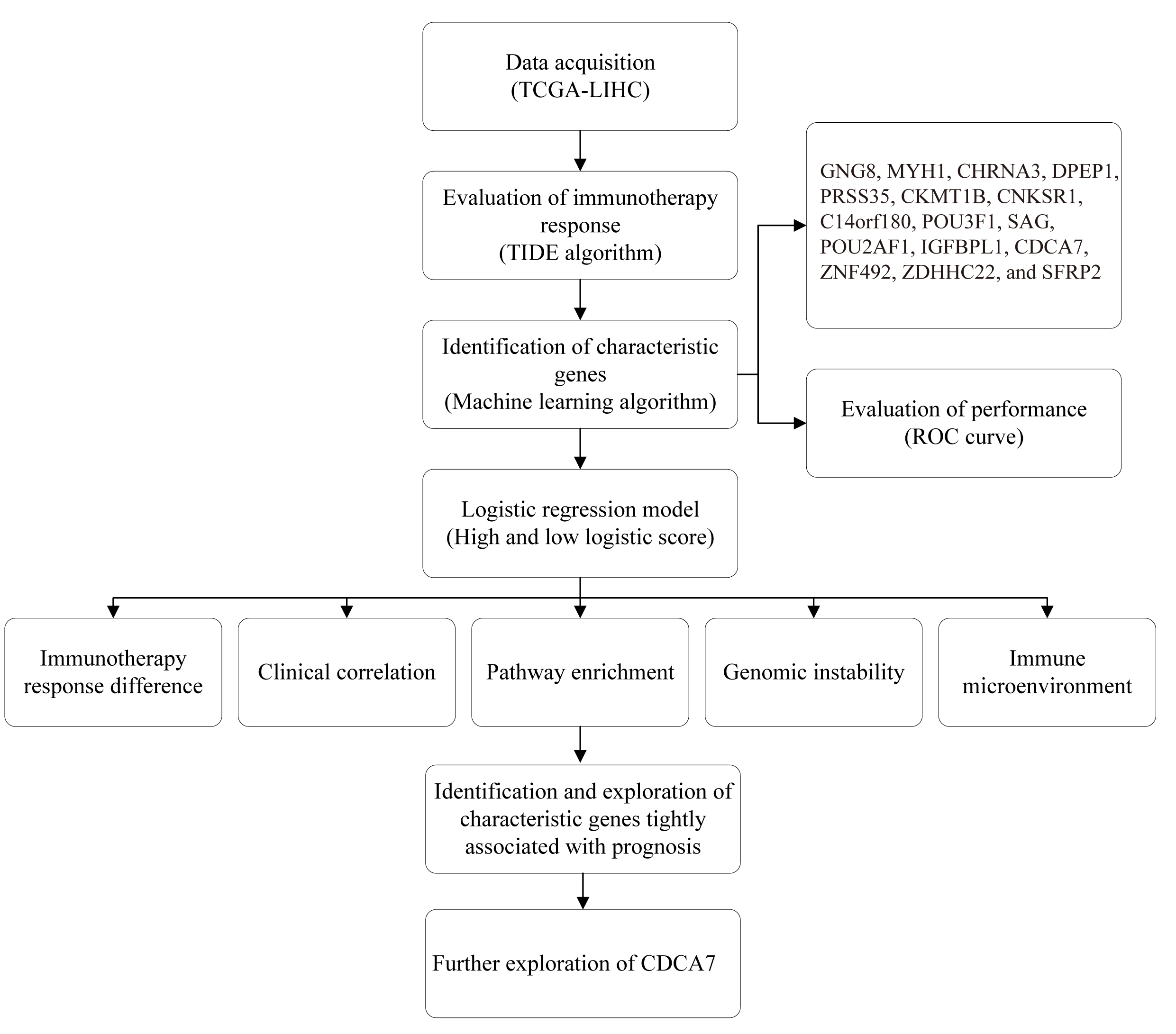

Supplement: Supplementary Figure 1 — The whole flow chart of the study. [file Image_1.tif]

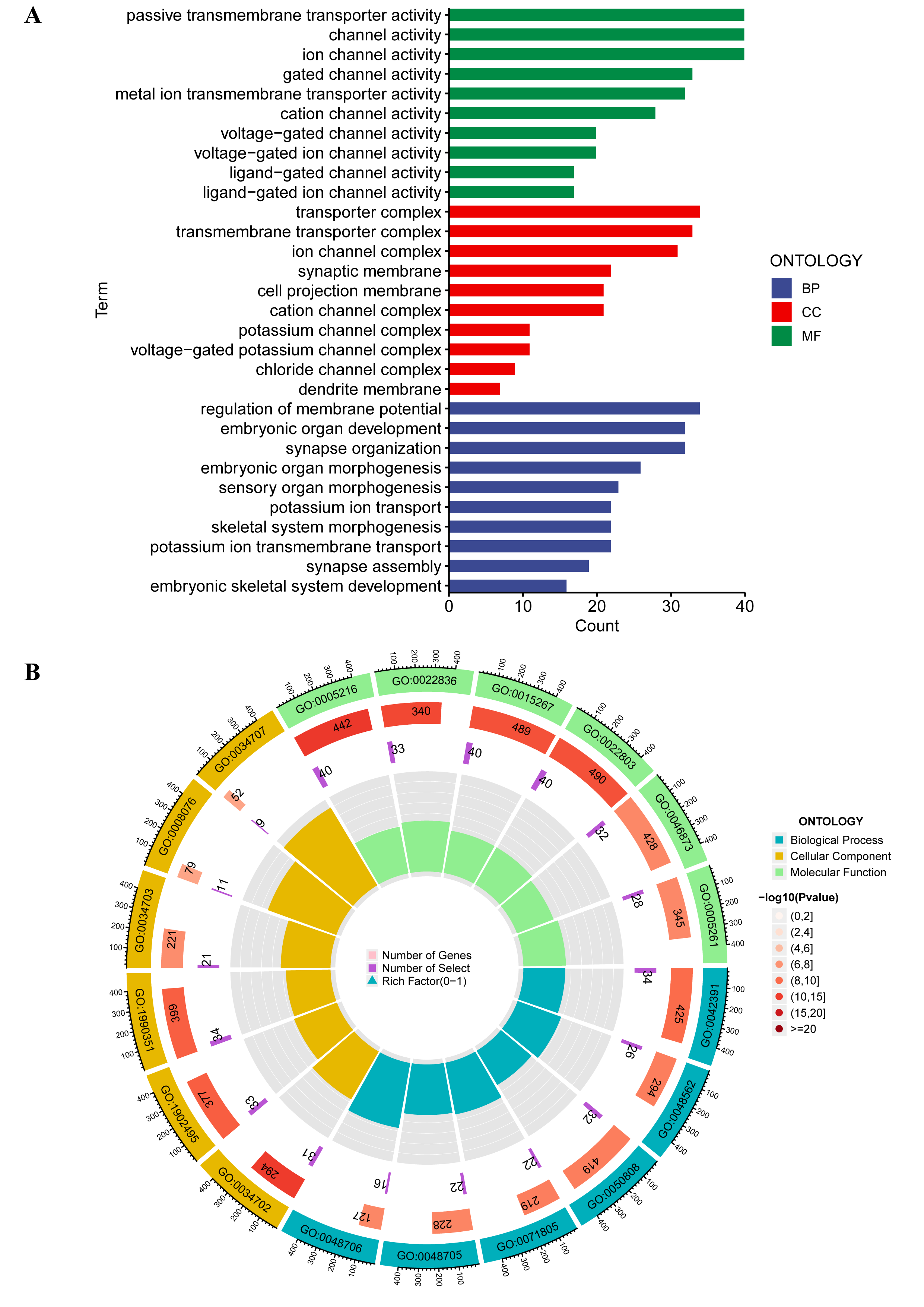

Supplement: Supplementary Figure 2 — GO enrichment analyses of DEGs. [file Image_2.tif]

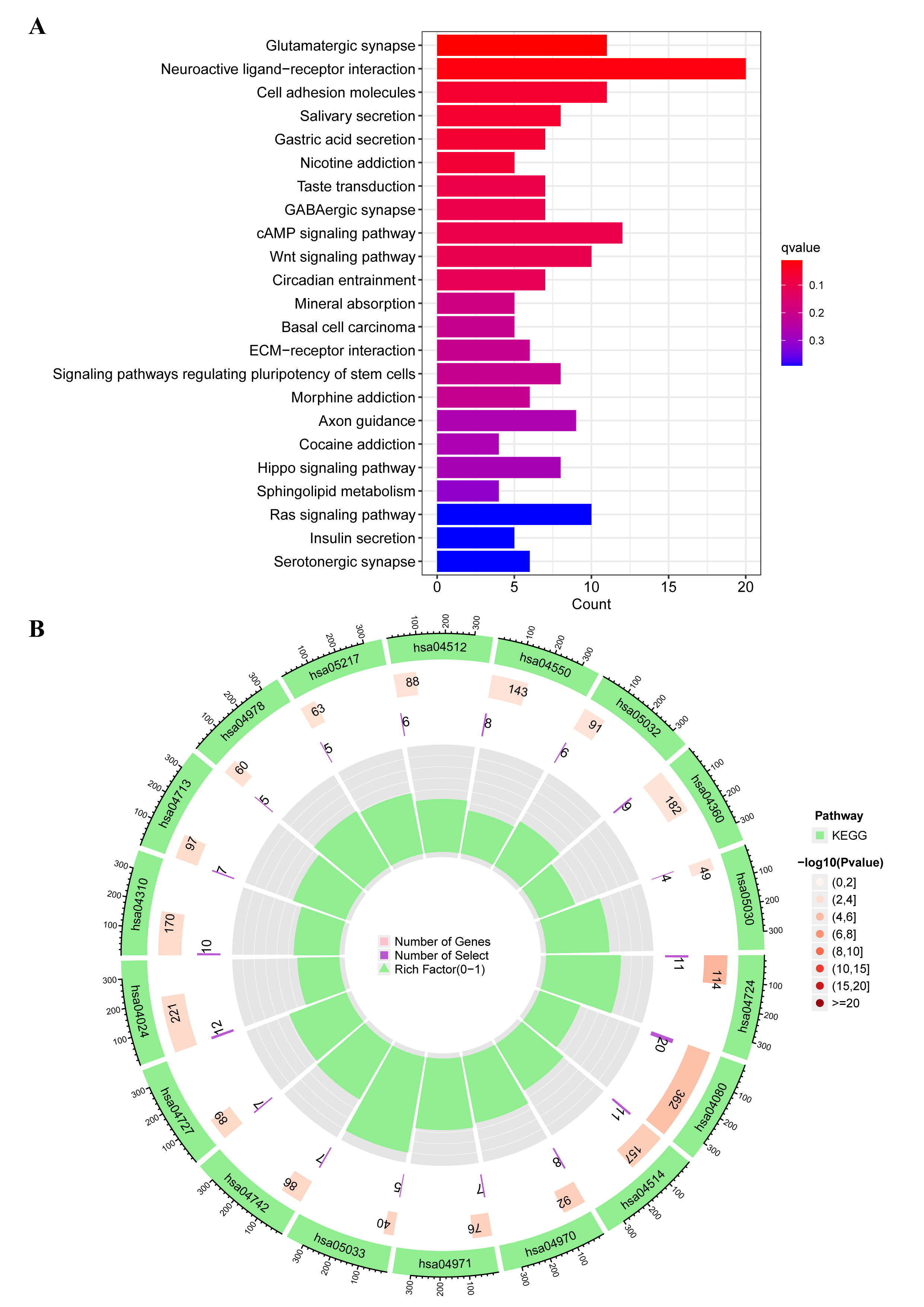

Supplement: Supplementary Figure 3 — KEGG enrichment analyses of DEGs. [file Image_3.tif]

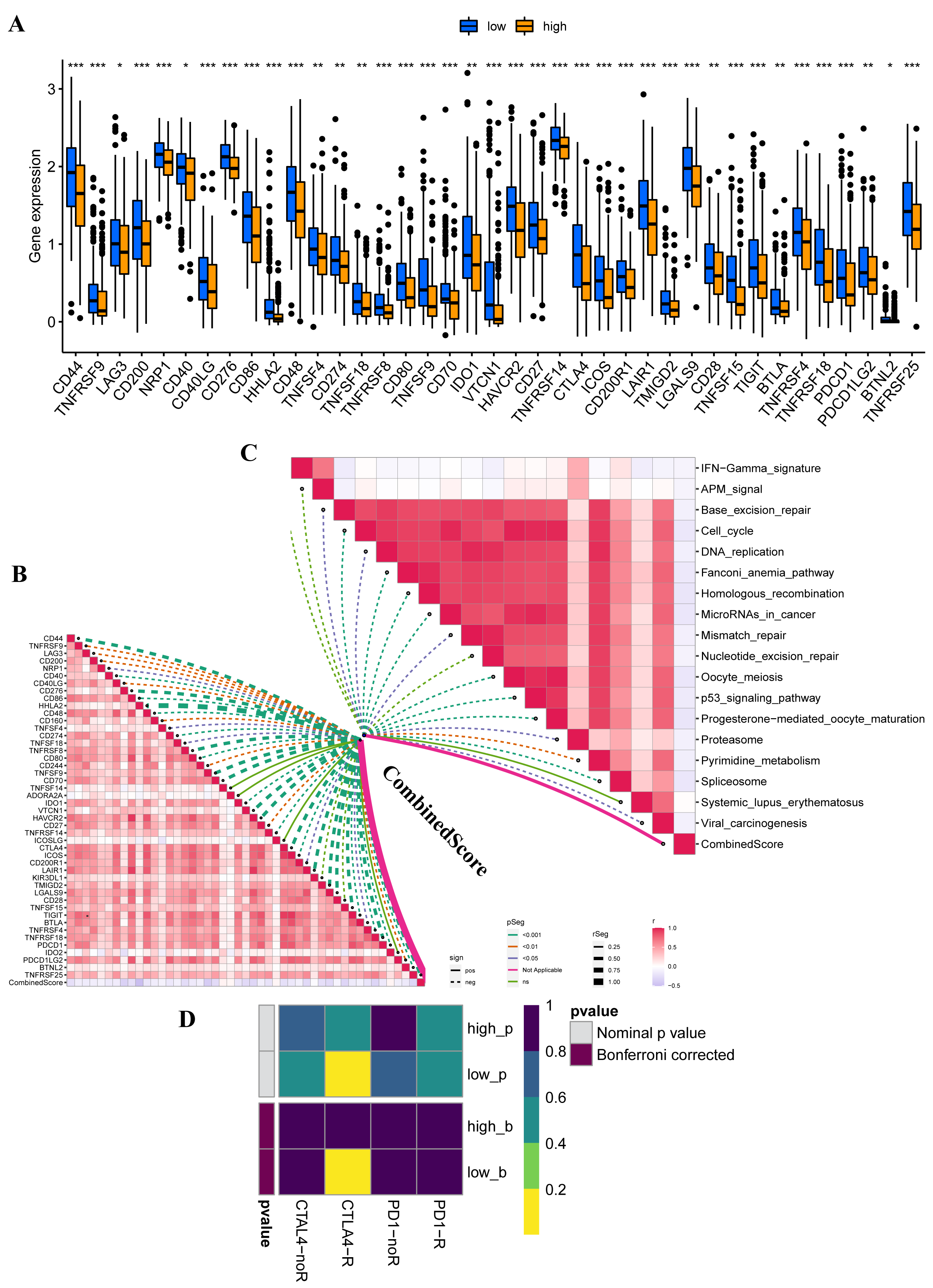

Supplement: Supplementary Figure 4 — (A) Expression of key immune checkpoints (PD-1, PD-L1, PD-L2, CTLA-4, etc.) in patients with a high- and low-CombinedScore in the combined ICGC cohort; (B) Correlations between the CombinedScore and expression of immune checkpoints in the combined ICGC cohort; (C) Correlations between the CombinedScore and immunotherapy predicted pathways scores in the combined ICGC cohort; (D) Submap algorithm was conducted to evaluate the sensitivity to PD-1 and CTLA-4 blockades in patients with a high- and low-CombinedScore in the combined ICGC cohort. [file Image_4.tif]

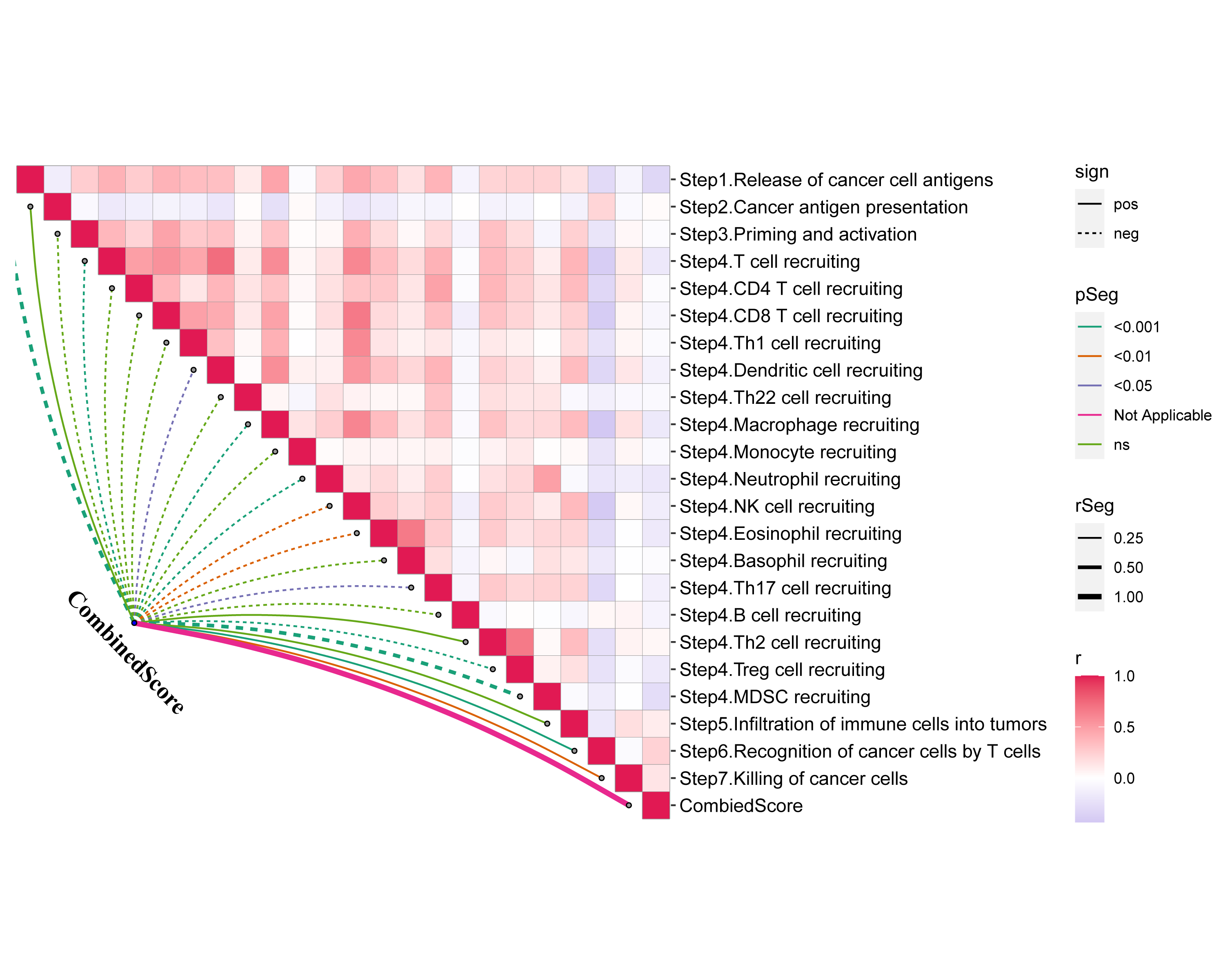

Supplement: Supplementary Figure 5 — Correlation analysis between CombinedScore and the activities of the anticancer immunity cycles. [file Image_5.tif]
